# Supplementary material for: The dental calculus metabolome in modern and historic samples
Source: Metabolomics. 2017 Oct 3;13(11):134. doi: 10.1007/s11306-017-1270-3 (PMC5626792; doi:10.1007/s11306-017-1270-3)
Supplement: Supplementary file 3 — Supplementary material 3 (DOCX 143 KB) [file 11306_2017_1270_MOESM3_ESM.docx]

**Table S3.** Differential abundance of UPLC-MS/MS-identified metabolites between modern and historic dental calculus^a^

| Metabolite | Mean relative frequency (%) | | q-value | Effect size**^b^** | CI 95% | |
| --- | --- | --- | --- | --- | --- | --- |
|  | Historic Modern | |  |  | Lower Upper | |
| 1,2-dipalmitoyl-GPC (16:0/16:0) | 0.28±0.12 | 0.21±0.16 | 0.052 | 0.078 | -0.068 | 0.238 |
| 1,2-dipalmitoyl-GPE (16:0/16:0)^d^ | 0.51±0.5 | 0.13±0.13 | 0.007 | 0.376 | 0.128 | 0.719 |
| 1-oleoyl-2-linoleoyl-GPC (18:1/18:2)^d^ | 0.55±0.48 | 0.05±0.04 | <0.001 | 0.522 | 0.139 | 0.905 |
| 1-palmitoyl-2-linoleoyl-GPC (16:0/18:2)^c^ | 0.82±0.56 | 0.05±0.04 | <0.001 | 0.764 | 0.466 | 1.115 |
| 1-palmitoyl-2-oleoyl-GPC (16:0/18:1)^c^ | 0.58±0.39 | 0.14±0.15 | 0.002 | 0.444 | 0.196 | 0.701 |
| 1-palmitoyl-2-oleoyl-GPE (16:0/18:1) | 0.04±0.04 | 0.05±0.06 | 0.097 | -0.005 | -0.066 | 0.043 |
| 1-palmitoyl-GPE (16:0) | 0.29±0.14 | 0.08±0.09 | 0.001 | 0.208 | 0.093 | 0.323 |
| 1-stearoyl-2-linoleoyl-GPC (18:0/18:2)^cd^ | 0.82±0.6 | 0.07±0.06 | <0.001 | 0.754 | 0.448 | 1.13 |
| 1-stearoyl-2-linoleoyl-GPE (18:0/18:2)^d^ | 0.54±0.16 | 0.18±0.28 | 0.006 | 0.351 | 0.057 | 0.557 |
| 1-stearoyl-2-oleoyl-GPC (18:0/18:1) | 0.34±0.19 | 0.1±0.13 | 0.003 | 0.234 | 0.078 | 0.393 |
| 1-stearoyl-GPC (18:0) | 0.4±0.07 | 0.09±0.08 | <0.001 | 0.307 | 0.228 | 0.374 |
| 1-stearoyl-GPE (18:0) | 0.45±0.13 | 0.12±0.1 | <0.001 | 0.327 | 0.209 | 0.433 |
| 10-hydroxystearate | 0.12±0.05 | 0.13±0.09 | 0.086 | -0.017 | -0.109 | 0.063 |
| 13-methylmyristate^c^ | 0.73±0.45 | 0.07±0.03 | <0.001 | 0.668 | 0.431 | 0.947 |
| 15-methylpalmitate^c^ | 0.85±0.57 | 0.05±0.03 | <0.001 | 0.803 | 0.519 | 1.154 |
| 17-methylstearate^c^ | 0.83±0.38 | 0.03±0.02 | <0.001 | 0.795 | 0.592 | 1.039 |
| 2'-deoxyguanosine | 0.45±0.17 | 0.63±1 | 0.084 | -0.186 | -1.207 | 0.424 |
| 2'-deoxyinosine | 0.01±0 | 0.08±0.09 | 0.022 | -0.066 | -0.146 | 0.005 |
| **2,3-dihydroxyisovalerate**^c†^ | **0.54±0.21** | **2.91±2.71** | **0.016** | **-2.366** | **-5.025** | **-0.485** |
| 2-hydroxy-3-methylvalerate | 0.07±0.02 | 0.06±0.04 | 0.067 | 0.012 | -0.02 | 0.045 |
| 2-hydroxyadipate | 0.81±0.37 | 0.02±0.01 | <0.001 | 0.791 | 0.598 | 1.018 |
| 2-hydroxyglutarate | 0.42±0.2 | 0.44±0.27 | 0.102 | -0.014 | -0.276 | 0.222 |
| **2-hydroxypalmitate**^c^**^*^** | **1.33±0.9** | **0.04±0.02** | **<0.001** | **1.299** | **0.808** | **1.857** |
| **2-hydroxystearate**^c^**^*^** | **1.43±1.01** | **0.01±0.01** | **<0.001** | **1.416** | **0.895** | **2.069** |
| 2-isopropylmalate | 0.45±0.18 | 1.35±1.1 | 0.02 | -0.899 | -1.986 | -0.032 |
| 3-hydroxy-3-methylglutarate^c^ | 0.59±0.25 | 0.11±0.11 | <0.001 | 0.48 | 0.298 | 0.657 |
| 3-hydroxybutyrate (BHBA) | 0.59±0.55 | 0.13±0.08 | 0.004 | 0.46 | 0.177 | 0.814 |
| 3-hydroxydecanoate | 0.35±0.2 | 0.13±0.09 | 0.002 | 0.221 | 0.094 | 0.375 |
| 3-hydroxylaurate | 0.48±0.26 | 0.36±0.3 | 0.065 | 0.117 | -0.225 | 0.399 |
| 3-hydroxymyristate^c^ | 0.52±0.23 | 0.09±0.05 | <0.001 | 0.435 | 0.314 | 0.593 |
| 3-hydroxypalmitate^c^ | 0.72±0.36 | 0.03±0.02 | <0.001 | 0.693 | 0.499 | 0.906 |
| 3-hydroxystearate^c^ | 0.74±0.29 | 0.04±0.03 | <0.001 | 0.697 | 0.548 | 0.873 |
| 3-methyl-2-oxobutyrate | 0.12±0.03 | 0.08±0.03 | 0.009 | 0.041 | 0.01 | 0.074 |
| 3-methyl-2-oxovalerate | 0.67±0.83 | 0.65±0.38 | 0.105 | 0.012 | -0.481 | 0.67 |
| 3-phenylpropionate (hydrocinnamate) | 0.02±0 | 0.22±0.11 | 0.001 | -0.202 | -0.3 | -0.109 |
| 3-phosphoglycerate | 0.11±0.09 | 0.15±0.09 | 0.054 | -0.046 | -0.144 | 0.05 |
| 3-sulfo-L-alanine^c^ | 0.6±0.28 | 0.07±0.02 | <0.001 | 0.534 | 0.378 | 0.701 |
| 4-acetamidobutanoate^c^ | 0.93±1.05 | 0.18±0.17 | 0.007 | 0.747 | 0.221 | 1.411 |
| 4-cholesten-3-one^c^ | 0.58±0.43 | 0.07±0.05 | 0.001 | 0.514 | 0.291 | 0.784 |
| **4-guanidinobutanoate**^c^**^*^** | **1.45±0.89** | **0.01±0.01** | **<0.001** | **1.431** | **0.907** | **1.932** |
| 4-hydroxy-2-oxoglutaric acid^c^ | 0.77±0.41 | 0.04±0.02 | <0.001 | 0.728 | 0.492 | 0.973 |
| 4-imidazoleacetate | 0.68±0.33 | 0.08±0.09 | <0.001 | 0.594 | 0.409 | 0.805 |
| 4-methyl-2-oxopentanoate | 0.04±0.01 | 0.06±0.03 | 0.03 | -0.022 | -0.052 | 0.006 |
| 5-aminovalerate | 0.43±0.26 | 0.55±0.12 | 0.037 | -0.116 | -0.285 | 0.074 |
| 5-oxoproline^c^ | 0.56±0.21 | 0.24±0.13 | 0.001 | 0.32 | 0.151 | 0.474 |
| 7-hydroxycholesterol (alpha or beta) | 0.85±0.5 | 0.004±0.001 | <0.001 | 0.847 | 0.59 | 1.137 |
| 7-ketocholesterol | 1±0.67 | 0.01±0.002 | <0.001 | 0.99 | 0.638 | 1.409 |
| adenine^c^ | 0.47±0.12 | 0.66±0.16 | 0.009 | -0.192 | -0.354 | -0.034 |
| adenosine^c^ | 0.63±0.32 | 0.87±0.33 | 0.031 | -0.246 | -0.597 | 0.08 |
| adenosine 2'-monophosphate (2'-AMP)^c^ | 0.55±0.35 | 0.35±0.2 | 0.03 | 0.194 | -0.061 | 0.463 |
| adenosine 5'-monophosphate (AMP) | 0.55±0.42 | 0.5±0.18 | 0.087 | 0.05 | -0.214 | 0.349 |
| adipate | 0.79±0.57 | 0.02±0.01 | <0.001 | 0.776 | 0.471 | 1.109 |
| **alanine**^c†^ | **0.66±0.59** | **5.49±1.01** | **<0.001** | **-4.827** | **-5.699** | **-3.873** |
| alpha-hydroxyisovalerate^c^ | 0.47±0.21 | 0.21±0.1 | 0.001 | 0.266 | 0.12 | 0.427 |
| alpha-ketoglutarate^c^ | 0.49±0.22 | 1.46±0.83 | 0.007 | -0.967 | -1.678 | -0.253 |
| alpha-tocopherol | 0.4±0.14 | 0.05±0.04 | <0.001 | 0.342 | 0.265 | 0.428 |
| arabonate/xylonate | 0.38±0.24 | 0.07±0.04 | <0.001 | 0.308 | 0.181 | 0.448 |
| arachidate (20:0)^c^ | 0.9±0.44 | 0.04±0.02 | <0.001 | 0.865 | 0.624 | 1.098 |
| asparagine | 0.58±0.2 | 0.04±0.02 | <0.001 | 0.542 | 0.153 | 0.93 |
| aspartate^c^ | 0.43±0.13 | 0.79±0.44 | 0.02 | -0.358 | -0.75 | 0.036 |
| azelate (nonanedioate) | 0.56±0.25 | 0.04±0.02 | <0.001 | 0.516 | 0.136 | 0.895 |
| behenoyl sphingomyelin (d18:1/22:0)^d^ | 0.34±0.14 | 0.05±0.03 | <0.001 | 0.292 | 0.212 | 0.38 |
| benzoate^c^ | 0.96±0.57 | 0.04±0.01 | <0.001 | 0.917 | 0.633 | 1.262 |
| betaine^c^ | 0.49±0.17 | 1.06±0.45 | 0.005 | -0.57 | -0.997 | -0.166 |
| caprate (10:0) | 0.81±0.47 | 0.04±0.01 | <0.001 | 0.775 | 0.554 | 1.088 |
| caproate (6:0) | 0.73±0.69 | 0.05±0.02 | 0.001 | 0.68 | 0.342 | 1.113 |
| caprylate (8:0) | 0.81±0.55 | 0.02±0.01 | <0.001 | 0.781 | 0.516 | 1.107 |
| ceramide (d18:1/14:0, d16:1/16:0)^d^ | 0.71±0.46 | 0.02±0.01 | <0.001 | 0.683 | 0.444 | 0.955 |
| chenodeoxycholate | 0.32±0.23 | 0.05±0.03 | <0.001 | 0.285 | -0.022 | 0.592 |
| cholate | 0.48±0.18 | 0.1±0.08 | <0.001 | 0.37 | 0.249 | 0.494 |
| cholesterol | 0.64±0.27 | 0.05±0.03 | <0.001 | 0.59 | 0.445 | 0.755 |
| choline^c^ | 0.47±0.31 | 0.27±0.12 | 0.016 | 0.197 | -0.003 | 0.43 |
| choline phosphate | 0.45±0.26 | 0.08±0.08 | <0.001 | 0.371 | 0.207 | 0.539 |
| citraconate/glutaconate^c^ | 0.89±0.35 | 0.03±0.01 | <0.001 | 0.866 | 0.695 | 1.067 |
| citrate^c^ | 0.46±0.34 | 0.24±0.13 | 0.015 | 0.221 | 0.002 | 0.447 |
| **citrulline**^c†^ | **0.49±0.15** | **2.65±1.42** | **0.002** | **-2.152** | **-3.347** | **-0.917** |
| cysteine s-sulfate | 0.2±0.04 | 0.07±0.06 | <0.001 | 0.126 | 0.072 | 0.179 |
| cytidine^c^ | 0.57±0.26 | 0.55±0.17 | 0.097 | 0.02 | -0.193 | 0.217 |
| cytidine 5'-monophosphate (5'-CMP) | 0.14±0.03 | 0.07±0.03 | 0.001 | 0.073 | 0.039 | 0.103 |
| cytosine | 0.55±0.1 | 0.84±0.74 | 0.059 | -0.289 | -0.931 | 0.303 |
| deoxycarnitine | 1.9x10^-4^ ±0.5x10^-4^ | 0.06±0.01 | <0.001 | -0.057 | -0.07 | -0.044 |
| deoxycholate | 0.34±0.41 | 0.07±0.07 | 0.01 | 0.271 | 0.08 | 0.578 |
| dimethylglycine | 0.31±0.06 | 0.05±0.02 | <0.001 | 0.251 | 0.212 | 0.289 |
| dipicolinate^c^ | 0.8±0.54 | 0.06±0.04 | <0.001 | 0.736 | 0.487 | 1.109 |
| dodecanedioate | 0.53±0.25 | 0.03±0.02 | <0.001 | 0.514 | 0.139 | 0.89 |
| eicosenoate (20:1)^c^ | 0.69±0.57 | 0.05±0.02 | 0.001 | 0.638 | 0.34 | 0.98 |
| erucate (22:1n9)^c^ | 0.81±0.59 | 0.04±0.02 | <0.001 | 0.765 | 0.466 | 1.123 |
| erythritol | 0.38±0.19 | 1.82±3.55 | 0.057 | -1.431 | -4.992 | 0.415 |
| erythronate^cd^ | 0.59±0.27 | 0.08±0.05 | <0.001 | 0.508 | 0.351 | 0.673 |
| fructose 1,6-diphosphate/glucose 1,6-diphosphate/myo-inositol diphosphates | 0.68±0.48 | 0.04±0.04 | <0.001 | 0.633 | 0.357 | 0.933 |
| fumarate | 0.56±0.24 | 1.02±0.21 | 0.001 | -0.458 | -0.71 | -0.251 |
| gamma-aminobutyrate (GABA) | 1.22±2.47 | 0.87±0.41 | 0.079 | 0.352 | -0.721 | 2.149 |
| gamma-glutamylglutamate | 0.27±0.18 | 0.39±0.2 | 0.044 | -0.117 | -0.339 | 0.085 |
| gluconate | 0.26±0.2 | 0.2±0.06 | 0.046 | 0.068 | -0.039 | 0.21 |
| **glucose**^†^ | **0.25±0.41** | **4.63±2.8** | **0.002** | **-4.376** | **-6.874** | **-1.65** |
| **glutamate**^c†^ | **0.49±0.17** | **3.56±0.84** | **<0.001** | **-3.063** | **-3.604** | **-2.216** |
| glutarate (pentanedioate)^c^ | 0.52±0.19 | 0.47±0.22 | 0.082 | 0.05 | -0.177 | 0.261 |
| glycerate^c^ | 0.52±0.29 | 0.15±0.09 | 0.001 | 0.369 | 0.187 | 0.547 |
| glycerol 3-phosphate^c^ | 0.61±0.2 | 0.06±0.02 | <0.001 | 0.558 | 0.448 | 0.676 |
| glycerophosphoglycerol | 0.25±0.14 | 0.61±0.29 | 0.005 | -0.363 | -0.631 | -0.122 |
| glycerophosphorylcholine (GPC) | 0.21±0.15 | 0.31±0.22 | 0.053 | -0.104 | -0.322 | 0.083 |
| glycine^c^ | 0.54±0.18 | 0.29±0.09 | 0.001 | 0.253 | 0.119 | 0.388 |
| guanine | 0.77±0.59 | 0.21±0.31 | 0.006 | 0.56 | 0.158 | 1.07 |
| guanosine^c^ | 0.61±0.33 | 0.55±0.27 | 0.084 | 0.063 | -0.275 | 0.334 |
| heptanoate (7:0) | 1.02±0.49 | 0.04±0.01 | <0.001 | 0.981 | 0.724 | 1.255 |
| imidazole lactate | 0.32±0.12 | 0.23±0.38 | 0.079 | 0.091 | -0.303 | 0.331 |
| imidazole propionate | 0.52±0.16 | 0.66±0.91 | 0.088 | -0.138 | -1.05 | 0.469 |
| indole-3-carboxylic acid^c^ | 0.63±0.16 | 0.44±0.58 | 0.066 | 0.195 | -0.382 | 0.574 |
| inosine | 0.4±0.42 | 0.74±0.25 | 0.012 | -0.343 | -0.649 | -0.005 |
| isoleucine | 0.46±0.23 | 1.44±0.66 | 0.002 | -0.986 | -1.596 | -0.421 |
| isovalerate | 0.07±0.06 | 0.13±0.04 | 0.011 | -0.053 | -0.097 | -0.003 |
| kynurenate | 0.5±0.29 | 0.1±0.06 | <0.001 | 0.405 | 0.236 | 0.586 |
| lactosyl-N-palmitoyl-sphingosine (d18:1/16:0) | 0.53±0.38 | 0.12±0.12 | 0.002 | 0.407 | 0.183 | 0.662 |
| laurate (12:0)^c^ | 0.77±0.31 | 0.04±0.02 | <0.001 | 0.725 | 0.568 | 0.912 |
| leucine | 0.37±0.11 | 0.72±0.36 | 0.013 | -0.344 | -0.687 | -0.039 |
| lignoceroyl sphingomyelin (d18:1/24:0) | 0.45±0.13 | 0.07±0.05 | <0.001 | 0.383 | 0.307 | 0.472 |
| malate | 0.59±0.53 | 0.31±0.13 | 0.019 | 0.284 | 0.01 | 0.649 |
| malonate^c^ | 0.77±0.34 | 0.08±0.08 | <0.001 | 0.683 | 0.462 | 0.882 |
| mannitol/sorbitol | 0.13±0.1 | 1.09±1.7 | 0.04 | -0.955 | -2.661 | 0.01 |
| margarate (17:0)^c^ | 0.74±0.4 | 0.04±0.02 | <0.001 | 0.696 | 0.489 | 0.932 |
| methionine^c^ | 0.58±0.12 | 0.31±0.19 | 0.004 | 0.273 | 0.093 | 0.448 |
| methionine sulfoxide | 0.005±0.001 | 0.11±0.05 | <0.001 | -0.107 | -0.154 | -0.066 |
| methylmalonate (MMA) | 0.55±0.29 | 0.12±0.12 | 0.001 | 0.43 | 0.242 | 0.637 |
| methylsuccinate^c^ | 0.62±0.3 | 0.09±0.05 | <0.001 | 0.534 | 0.348 | 0.706 |
| myristate (14:0)^c^ | 0.85±0.6 | 0.01±0.002 | <0.001 | 0.847 | 0.601 | 1.241 |
| N-acetylaspartate (NAA)^c^ | 0.52±0.27 | 0.59±0.22 | 0.079 | -0.064 | -0.298 | 0.181 |
| N-acetylputrescine | 0.002±0.0006 | 0.1±0.06 | 0.003 | -0.093 | -0.156 | -0.044 |
| N-acetylserine | 0.05±0.01 | 0.07±0.04 | 0.054 | -0.019 | -0.059 | 0.013 |
| N-acetyltaurine | 0.32±0.12 | 0.1±0.06 | <0.001 | 0.222 | 0.131 | 0.309 |
| N-acetylthreonine | 0.09±0.02 | 0.06±0.02 | 0.004 | 0.028 | 0.009 | 0.047 |
| N-delta-acetylornithine | 0.07±0.07 | 0.28±0.16 | 0.004 | -0.212 | -0.368 | -0.065 |
| N-formylanthranilic acid^c^ | 0.7±0.24 | 0.03±0.01 | <0.001 | 0.662 | 0.532 | 0.802 |
| N-methylproline | 0.29±0.08 | 0.15±0.11 | 0.005 | 0.145 | 0.034 | 0.25 |
| N-palmitoyl-sphinganine (d18:0/16:0)^c^ | 0.91±0.38 | 0.01±0.01 | <0.001 | 0.898 | 0.69 | 1.105 |
| N-palmitoyl-sphingosine (d18:1/16:0)^c^ | 0.79±0.48 | 0.02±0.01 | <0.001 | 0.769 | 0.523 | 1.044 |
| N-stearoyl-sphingosine (d18:1/18:0)^d^ | 0.6±0.54 | 0.01±0.002 | 0.001 | 0.592 | 0.329 | 0.957 |
| **nicotinate**^c†^ | **0.9±1.25** | **11.1±3.54** | **<0.001** | **-10.19** | **-13.305** | **-6.906** |
| **nonadecanoate (19:0)**^c^**^*^** | **1.04±0.61** | **0.04±0.01** | **<0.001** | **1.001** | **0.68** | **1.397** |
| oleate/vaccenate (18:1)^c^ | 0.55±0.45 | 0.07±0.03 | 0.001 | 0.477 | 0.255 | 0.737 |
| **orotate**^c†^ | **0.56±0.32** | **7.11±3.71** | **0.001** | **-6.549** | **-9.731** | **-3.321** |
| oxalate (ethanedioate)^c^ | 0.71±0.25 | 0.03±0.01 | <0.001 | 0.684 | 0.555 | 0.819 |
| palmitate (16:0)^c^ | 0.64±0.19 | 0.04±0.01 | <0.001 | 0.595 | 0.489 | 0.7 |
| palmitoleate (16:1n7)^c^ | 0.55±0.28 | 0.08±0.05 | <0.001 | 0.47 | 0.31 | 0.632 |
| palmitoyl dihydrosphingomyelin (d18:0/16:0)^d^ | 0.51±0.31 | 0.02±0.02 | <0.001 | 0.514 | 0.141 | 0.886 |
| palmitoyl ethanolamide^c^ | 0.87±0.45 | 0.03±0.01 | <0.001 | 0.839 | 0.601 | 1.109 |
| palmitoyl sphingomyelin (d18:1/16:0) ^c^ | 0.52±0.31 | 0.09±0.07 | <0.001 | 0.434 | 0.248 | 0.619 |
| **pantothenate**^c†^ | **0.52±0.15** | **1.89±0.63** | **<0.001** | **-1.375** | **-1.913** | **-0.801** |
| pelargonate (9:0) | 0.67±0.37 | 0.02±0.01 | <0.001 | 0.648 | 0.456 | 0.886 |
| pentadecanoate (15:0) ^c^ | 0.58±0.19 | 0.04±0.01 | <0.001 | 0.535 | 0.425 | 0.647 |
| phenol sulfate | 0.14±0.03 | 0.06±0.06 | 0.017 | 0.088 | -0.132 | 0.308 |
| **phenylalanine**^c†^ | **0.53±0.16** | **2.74±1.5** | **0.002** | **-2.211** | **-3.491** | **-0.879** |
| phosphate^c^ | 0.64±0.18 | 0.04±0.01 | <0.001 | 0.593 | 0.493 | 0.699 |
| phosphoethanolamine | 0.3±0.41 | 0.14±0.17 | 0.044 | 0.155 | -0.101 | 0.444 |
| phytosphingosine | 0.72±0.74 | 0.05±0.03 | 0.002 | 0.674 | 0.375 | 1.128 |
| **pipecolate**^c†^ | **0.56±0.33** | **3.2±1.15** | **<0.001** | **-2.639** | **-3.588** | **-1.685** |
| **proline**^c†^ | **0.5±0.15** | **3.02±1.2** | **<0.001** | **-2.52** | **-3.528** | **-1.433** |
| pyridoxate | 0.26±0.06 | 0.11±0.09 | 0.002 | 0.15 | 0.065 | 0.229 |
| pyruvate | 0.42±0.21 | 0.3±0.13 | 0.031 | 0.119 | -0.055 | 0.296 |
| **salicylate^*^** | **1.1±0.84** | **0.03±0.01** | **<0.001** | **1.075** | **0.667** | **1.588** |
| sebacate (decanedioate) | 0.4±0.2 | 0.02±0.01 | <0.001 | 0.405 | 0.067 | 0.744 |
| serine^c^ | 0.46±0.21 | 0.95±0.49 | 0.012 | -0.493 | -0.942 | -0.079 |
| spermidine | 0.004±0.001 | 0.12±0.06 | 0.001 | -0.112 | -0.168 | -0.061 |
| **sphinganine**^c^**^*^** | **1.29±0.95** | **0.04±0.02** | **<0.001** | **1.25** | **0.731** | **1.765** |
| sphingomyelin (d18:1/14:0, d16:1/16:0)^d^ | 0.36±0.12 | 0.05±0.02 | <0.001 | 0.313 | 0.246 | 0.384 |
| sphingomyelin (d18:1/18:1, d18:2/18:0) | 0.51±0.09 | 0.06±0.02 | <0.001 | 0.451 | 0.398 | 0.499 |
| sphingomyelin (d18:1/24:1, d18:2/24:0)^d^ | 0.34±0.12 | 0.09±0.06 | <0.001 | 0.252 | 0.17 | 0.338 |
| sphingomyelin (d18:2/16:0, d18:1/16:1)^d^ | 0.28±0.05 | 0.04±0.02 | 0.001 | 0.229 | -0.056 | 0.514 |
| sphingomyelin (d18:2/24:1, d18:1/24:2)^d^ | 0.27±0.06 | 0.07±0.05 | <0.001 | 0.202 | 0.148 | 0.249 |
| sphingosine^c^ | 0.86±0.77 | 0.1±0.07 | 0.002 | 0.754 | 0.357 | 1.232 |
| **stachydrine**^†^ | **0.32±0.33** | **5.68±2.52** | **<0.001** | **-5.36** | **-7.807** | **-3.327** |
| stearate (18:0)^c^ | 0.71±0.3 | 0.04±0.02 | <0.001 | 0.667 | 0.525 | 0.868 |
| stearoyl ethanolamide^c^ | 0.92±0.44 | 0.04±0.01 | <0.001 | 0.872 | 0.654 | 1.138 |
| stearoyl sphingomyelin (d18:1/18:0) | 0.44±0.29 | 0.04±0.02 | <0.001 | 0.428 | 0.074 | 0.783 |
| succinate^c^ | 0.5±0.15 | 0.31±0.18 | 0.013 | 0.191 | -0.003 | 0.356 |
| **sulfate^d*^** | **1.38±1.13** | **0.005±0.002** | **<0.001** | **1.371** | **0.776** | **2.06** |
| taurine | 0.04±0.02 | 0.24±0.27 | 0.022 | -0.205 | -0.471 | -0.037 |
| threonate^c^ | 0.57±0.33 | 0.06±0.02 | <0.001 | 0.508 | 0.333 | 0.698 |
| **threonine**^†^ | **0.41±0.33** | **1.97±1.39** | **0.008** | **-1.556** | **-2.778** | **-0.387** |
| thymidine | 0.4±0.14 | 0.56±0.56 | 0.073 | -0.161 | -0.682 | 0.298 |
| trans-nonadecenoate (tr 19:1)^cd^ | 0.92±0.66 | 0.07±0.06 | <0.001 | 0.854 | 0.495 | 1.238 |
| trans-urocanate^c^ | 0.57±0.34 | 1.37±0.8 | 0.012 | -0.804 | -1.51 | -0.085 |
| **tyrosine**^c†^ | **0.5±0.09** | **2.11±1.28** | **0.005** | **-1.613** | **-2.712** | **-0.523** |
| uridine^c^ | 0.54±0.19 | 0.58±0.17 | 0.082 | -0.043 | -0.23 | 0.139 |
| valerate | 0.44±0.35 | 0.07±0.02 | 0.001 | 0.38 | 0.226 | 0.59 |
| **xanthine**^c†^ | **0.54±0.12** | **3.9±1.78** | **0.001** | **-3.36** | **-4.956** | **-1.849** |

^a^Significantly differentially abundant metabolites (q ≤ 0.05, effect size ≥ 1) between historic and modern calculus are in bold.

^b^Difference in Proportions (DP).

^c^Metabolites universally present in all modern and all historic calculus samples.

^d^ Indicates compounds confidently identified but that have not been officially confirmed based on a standard.

^*^Metabolites with significantly higher proportion in historic calculus.

^†^Metabolites with significantly higher proportion in modern calculus.
