# Supplementary material for: The dental calculus metabolome in modern and historic samples
Source: Metabolomics. 2017 Oct 3;13(11):134. doi: 10.1007/s11306-017-1270-3 (PMC5626792; doi:10.1007/s11306-017-1270-3)
Supplement: Supplementary file 4 — Supplementary material 4 (DOCX 11820 KB) [file 11306_2017_1270_MOESM4_ESM.docx]

Supplementary Materials

**The dental calculus metabolome in modern and historic samples**

1. **Supplementary Data**  2

**Figures S1-S8**  2-10

**Table S4 S8**  11

2. **Supplementary Materials and Methods** 12

*2.1 Calculus collection and preparation* 12

*2.2 Genetic Authentication of a Preserved Oral Microbiome in Historic Samples* 12

*2.3 Sample Preparation for Mass Spectrometry at Metabolon, Inc*. 12

*2.4 QA/QC at Metabolon, Inc.* 12

*2.5 Ultrahigh Performance Liquid Chromatography-Tandem Mass Spectroscopy*

*(UPLC-MS/MS) at Metabolon, Inc.* 13

*2.6 Data Extraction, Compound Identification, Quantification, and Normalization at Metabolon, Inc.*  13

*2.7 Further characterization of historic calculus by GC-MS and UPLC-MS/MS* 14

3. **References** 15

1. Supplementary Figures


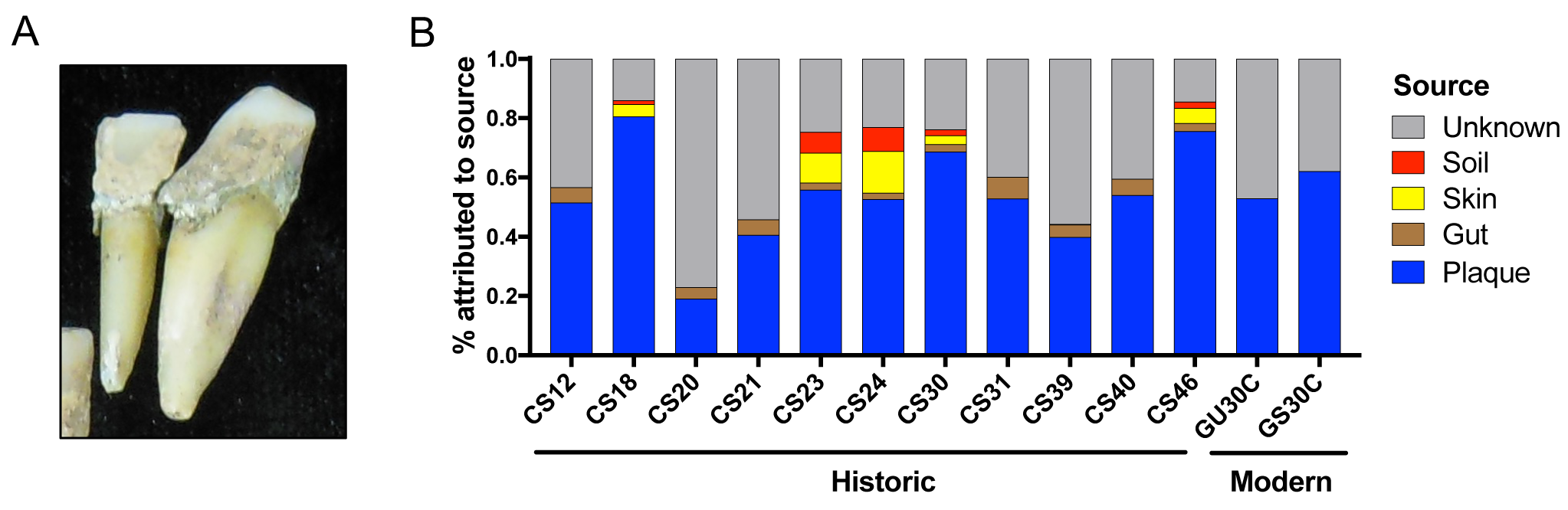


**Fig. S1** Historic calculus samples contain oral bacterial community profiles. **a** Historic calculus on teeth from CS18 prior to sampling. **b** Percent of bacterial community in calculus samples attributable to distinct environmental sources by SourceTracker analysis. Modern samples from (Ziesemer et al. 2015), demonstrate that a high proportion of the microbial community assigned to an “Unknown” source is characteristic of dental calculus. CS6 failed to build DNA libraries with the AccuPrimePFX polymerase.

**Fig. S2** Comparison of quantified metabolites from samples analyzed by Metabolon, Inc. and Wisconsin-Madison. Five calculus samples were analyzed by GC-MS and LC-MS in Madison, Wisconsin; the relative quantitation obtained on these methods was significantly correlated with results from Metabolon, Inc. (linear regression, p < 0.001).


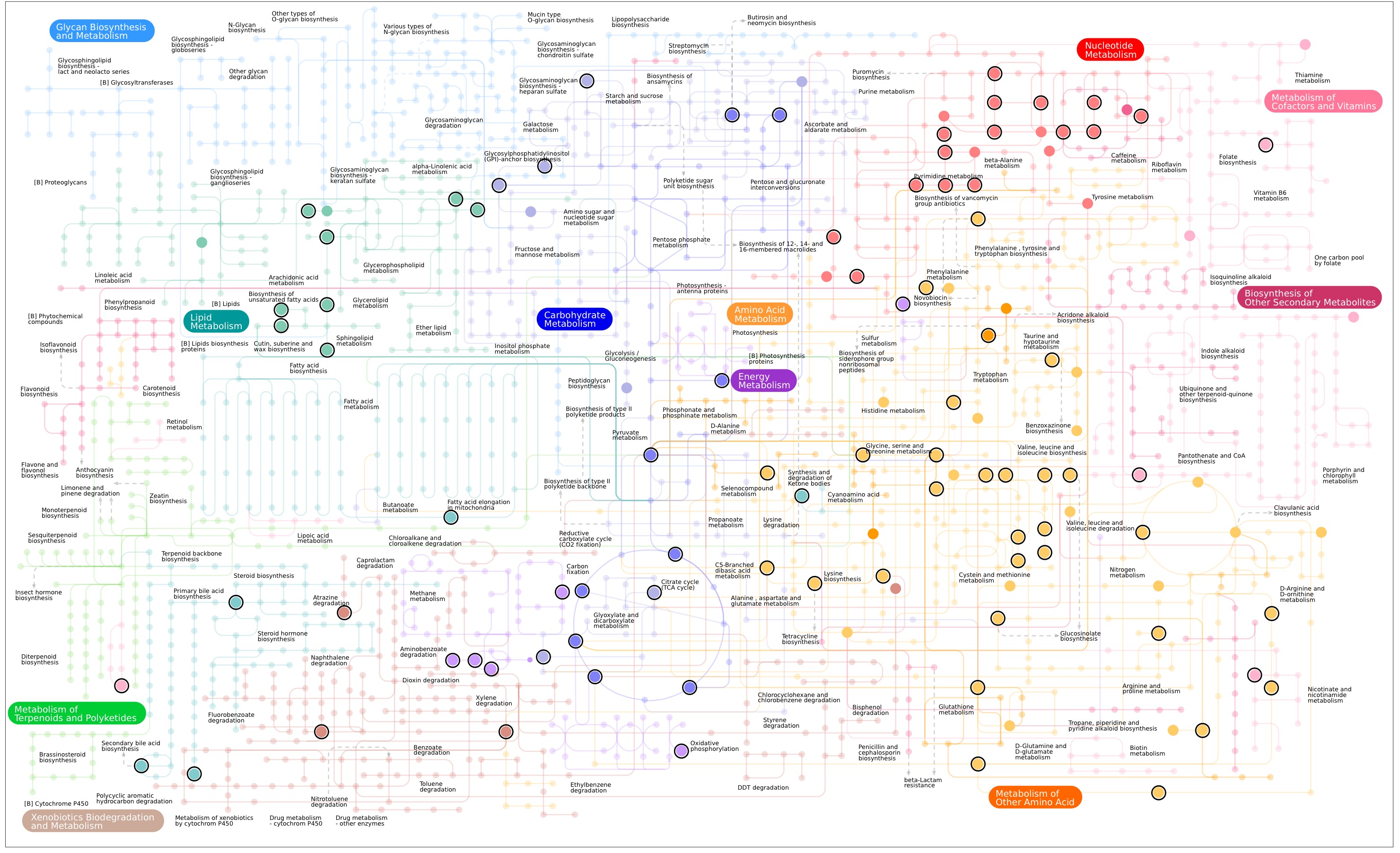


**Fig. S3**. Representation of diverse metabolic pathways preserved in calculus. Large circles represent metabolites identified in calculus. Black rings around large circles indicate the metabolite was detected in historic calculus.


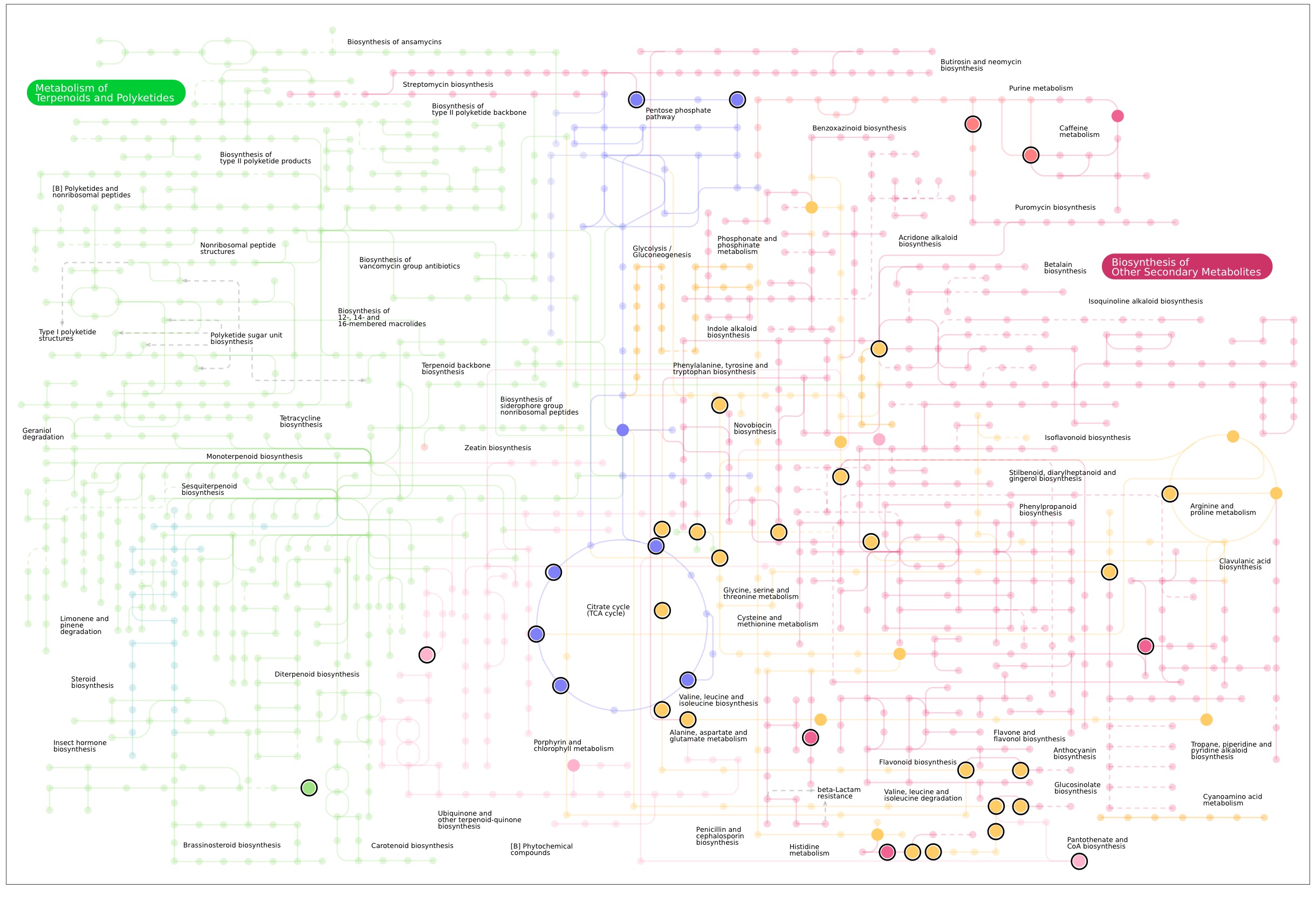


**Fig. S4**. Representation of pathways for biosynthesis of secondary metabolites preserved in calculus. Large circles represent metabolites identified in calculus. Black rings around large circles indicate the metabolite was detected in historic calculus.


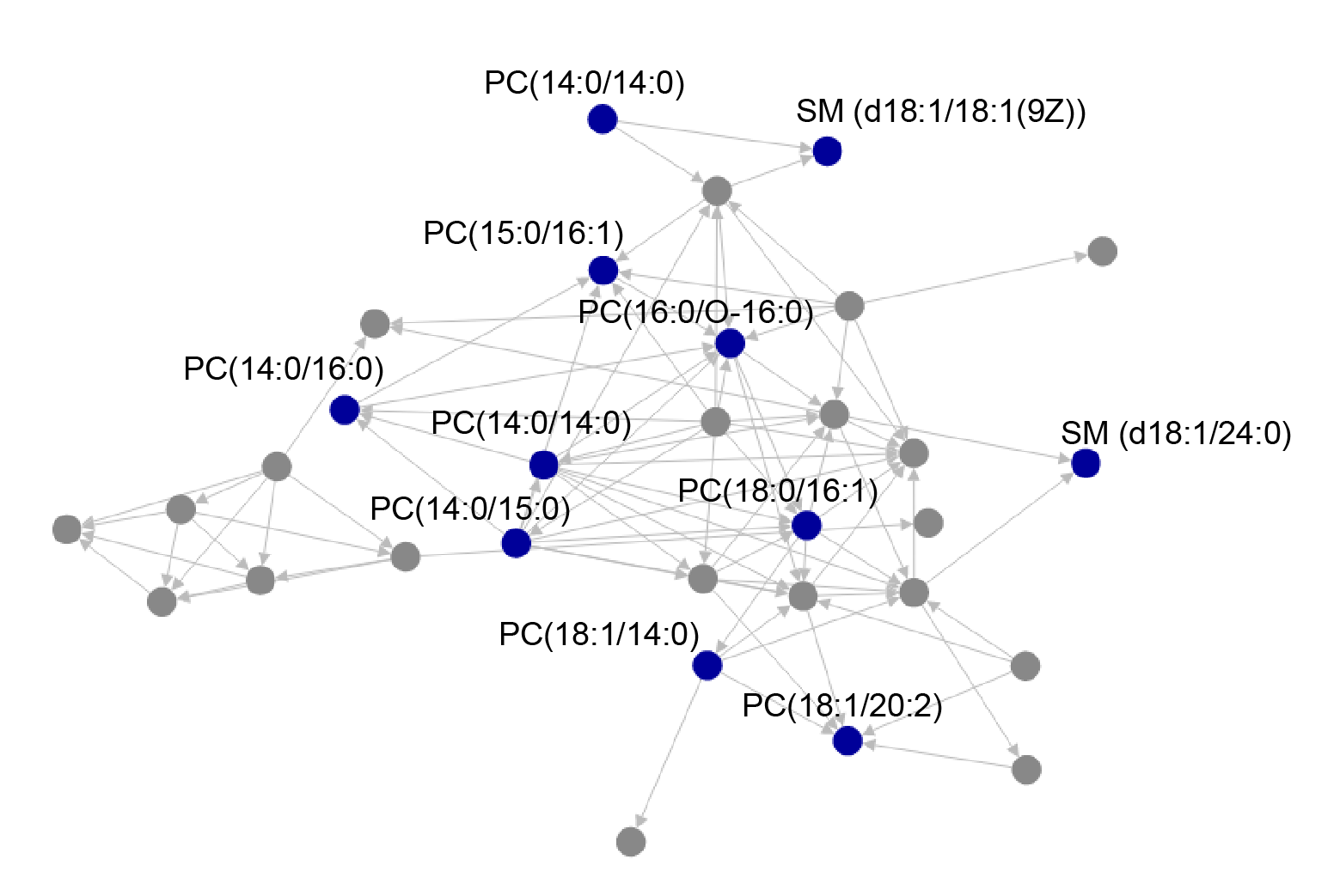


**Fig. S5** Global Natural Products Social Molecular Networking (GNPS) analysis of UPLC-MS/MS spectra (Wisconsin subset) clusters compounds containing phosphocholine head groups. This network of molecular clusters (nodes) contains molecules with putative identifications (dark blue) to phosphatidylcholines and sphingomyelins containing phosphocholine. The edges denote at least 3 matching fragments between spectra and a cosine score of >0.8.

**Fig. S6** Differences exist in mean proportions of metabolites universally detected in all historic and modern dental calculus samples. **a** Principal components analysis distinctly separates modern and historic calculus samples. **b** Metabolites with significant differences (q ≤ 0.05, effect size of ≥1.0) in mean proportions between historic and modern calculus.

**
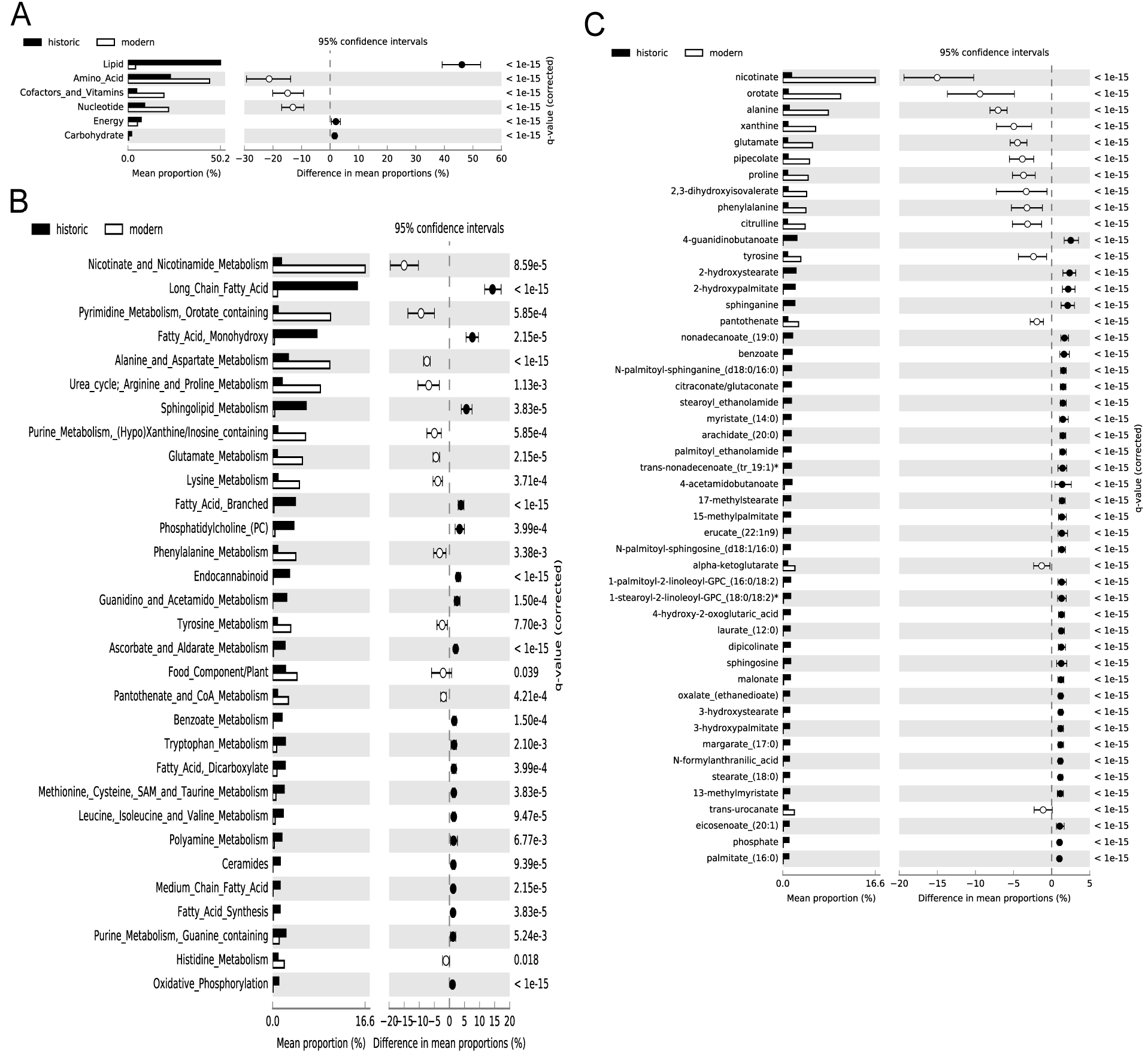
**

**Fig. S7** Differences exist in proportions of super-pathways, sub-pathways, and metabolites universally detected in all historic and modern dental calculus samples. Significantly different (q ≤ 0.05, effect size of ≥1.0) proportions of **a** Super-pathways, **b** Sub-pathways, and **c** Individual metabolites between historic and modern samples.

**
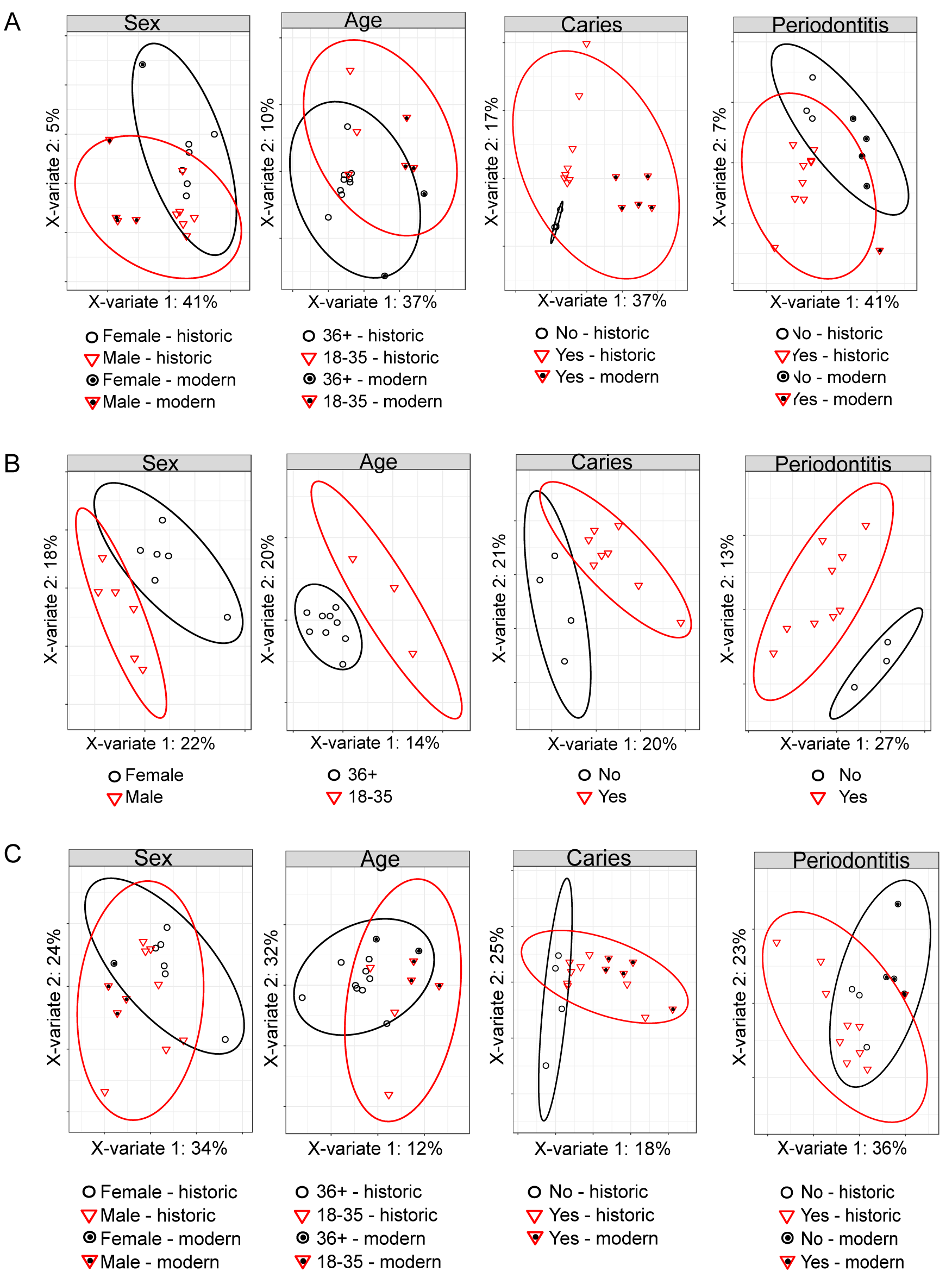
**

**Fig. S8** Partial least squares discriminant analysis of metabolites universally present in calculus samples. **a** Calculus samples cluster based on time period rather than biological category (sex, age, caries status and periodontal disease status) when including metabolites detected in all seventeen calculus samples. **b** Historic calculus samples cluster based on biological category (sex, age, caries status and periodontal disease status) when including metabolites detected in all twelve historic samples. **c** When using only universally detected metabolites in the *Lipid* and *Energy* categories (pathways with the best representation in historic samples), it was still not possible to discriminate samples based on biological category, although the separation between historic and modern samples was reduced slightly compared to **a**. Ellipses indicate 95% confidence intervals.

**Fig. S9** Comparison of sulfate abundance and the relative abundance of the oral sulfate-producing genus, *Methanobrevibacter*, shown using **a** linear and **b** log scales. *Methanobrevibacter* relative abundance was estimated using 16S rRNA gene sequence counts, and no correlation was observed with sulfate abundance. *Methanobrevibacter* was not detected in CS18.

**Table S4.** Q2 values for PLS-DAs

|  |  | Q2 | |
| --- | --- | --- | --- |
| Samples | Category | Component 1 | Component 2 |
| *Historic+Modern* |  |  |  |
| All detected metabolites^a^ | sex | 0.0083 | -1.8634 |
|  | age | -0.2584 | -0.6265 |
|  | caries | 0.0447 | -1.2258 |
|  | periodontitis | 0.0140 | -1.4872 |
| Universally detected metabolites^b^ | sex | -0.0716 | -2.3265 |
|  | age | -0.3976 | -1.2707 |
|  | caries | 0.0076 | -0.6977 |
|  | periodontitis | -0.0414 | -3.3357 |
| Energy+lipid metabolites^c^ | sex | -0.2627 | -0.6783 |
|  | age | -1.2403 | -0.8241 |
|  | caries | -0.1927 | -0.2627 |
|  | periodontitis | 0.0351 | -0.3423 |
| *Historic* |  |  |  |
| All detected metabolites^a^ | sex | -0.3503 | -1.0893 |
|  | age | -0.3200 | -3.3039 |
|  | caries | -0.1892 | -2.6131 |
|  | periodontitis | -0.1119 | -1.9819 |
| Universally detected metabolites^b^ | sex | -0.3232 | -1.2174 |
|  | age | -0.6185 | -2.2217 |
|  | caries | -0.3780 | -0.8847 |
|  | periodontitis | -0.1054 | -1.7231 |

^a^Includes all metabolites detected in at least one sample.

^b^Includes metabolites detected in all samples.

^c^Includes all energy and lipid metabolites detected in at least one sample.

**2 Supplementary Materials and Methods**

*2.1 Calculus collection and preparation*

All of the skeletons were from earth cut graves and had either been contained within wooden coffins, subsequently decomposed, or had been buried in shrouds uncoffined. The surfaces of the teeth were cleaned with 5% NaOCl followed by water prior to sampling to remove traces of burial soil, and sampling was performed wearing gloves and a mask over the nose and mouth. Calculus samples were collected in individual tubes on site, and removed to the Research Laboratory for Archaeology and the History of Art at the University of Oxford where ~20 mg was subsampled, placed in a new tube and crushed by micropestle. Crushed historic calculus samples were sent for metabolomics analyses without further processing.

*2.2 Genetic Authentication of a Preserved Oral Microbiome in Historic Samples*

Shotgun Illumina libraries were constructed following previously described methods (Meyer and Kircher 2010) with AccuPrime PFX polymerase (Invitrogen), and sequenced on an Illumina HiSeq2500 at the University of Copenhagen National High-Throughput DNA Sequencing Centre. Prior to analysis, reads were de-mulitplexed, quality-checked, and trimmed of adapters using AdapterRemoval v.1 (Lindgreen 2012) with the following non-default parameters: --maxns 0, --trimns, --trimqualities --minquality 30, --minlength 25, --collapse, and --minalignmentlength 10. To identify 16S rRNA gene reads in the metagenomic dataset, reads were aligned to the Greengenes v. 13.8 database using bowtie2 (Langmead and Salzberg 2012).

*2.3 Sample Preparation for Mass Spectrometry at Metabolon, Inc.*

Samples (~20 mg) were decalcified in 0.5M EDTA, centrifuged to pellet debris, and supernatant prepared using the automated MicroLab STAR® system from Hamilton Company. Several recovery standards were added prior to the first step in the extraction process for QC purposes. To remove protein, dissociate small molecules bound to protein or trapped in the precipitated protein matrix, and to recover chemically diverse metabolites, proteins were precipitated with methanol under vigorous shaking for 2 min (Glen Mills GenoGrinder 2000) followed by centrifugation. The resulting extract was divided into five fractions: two for analysis by two separate reverse phase (RP)/UPLC-MS/MS methods with positive ion mode electrospray ionization (ESI), one for analysis by RP/UPLC-MS/MS with negative ion mode ESI, one for analysis by HILIC/UPLC-MS/MS with negative ion mode ESI, and one sample was reserved for backup. Samples were placed briefly on a TurboVap® (Zymark) to remove the organic solvent. The sample extracts were stored overnight under nitrogen before preparation for analysis.

*2.4 QA/QC at Metabolon, Inc.*

Several types of controls were analyzed in concert with the experimental samples: a pooled matrix sample generated by taking a small volume of each experimental sample (or alternatively, use of a pool of well-characterized human plasma) served as a technical replicate throughout the data set; extracted water samples served as process blanks; and a cocktail of QC standards that were carefully chosen not to interfere with the measurement of endogenous compounds were spiked into every analyzed sample, allowed instrument performance monitoring and aided chromatographic alignment. Instrument variability was determined by calculating the median relative standard deviation (RSD) for the standards that were added to each sample prior to injection into the mass spectrometers. Overall process variability was determined by calculating the median RSD for all sample metabolites (i.e., non-instrument standards) present in 100% of the pooled matrix samples. Experimental samples were randomized across the platform run with QC samples spaced evenly among the injections.

*2.5 Ultrahigh Performance Liquid Chromatography-Tandem Mass Spectroscopy (UPLC-MS/MS) at Metabolon, Inc.*

All methods utilized a Waters ACQUITY ultra-performance liquid chromatography (UPLC) and a Thermo Scientific Q-Exactive high resolution/accurate mass spectrometer interfaced with a heated electrospray ionization (HESI-II) source and Orbitrap mass analyzer operated at 35,000 mass resolution. The sample extract was dried then reconstituted in solvents compatible to each of the four methods. Each reconstitution solvent contained a series of standards at fixed concentrations to ensure injection and chromatographic consistency. One aliquot was analyzed using acidic positive ion conditions, chromatographically optimized for more hydrophilic compounds. In this method, the extract was gradient eluted from a C18 column (Waters UPLC BEH C18-2.1x100 mm, 1.7 µm) using water and methanol, containing 0.05% perfluoropentanoic acid (PFPA) and 0.1% formic acid (FA). Another aliquot was also analyzed using acidic positive ion conditions, however it was chromatographically optimized for more hydrophobic compounds. In this method, the extract was gradient eluted from the same afore mentioned C18 column using methanol, acetonitrile, water, 0.05% PFPA and 0.01% FA and was operated at an overall higher organic content. Another aliquot was analyzed using basic negative ion optimized conditions using a separate dedicated C18 column. The basic extracts were gradient eluted from the column using methanol and water, however with 6.5mM Ammonium Bicarbonate at pH 8. The fourth aliquot was analyzed via negative ionization following elution from a HILIC column (Waters UPLC BEH Amide 2.1x150 mm, 1.7 µm) using a gradient consisting of water and acetonitrile with 10mM Ammonium Formate, pH 10.8. The MS analysis alternated between MS and data-dependent MSn scans using dynamic exclusion. The scan range varied slighted between methods but covered 70-1000 m/z. Raw data files are archived and extracted as described below.

*2.6 Data Extraction, Compound Identification, Quantification, and Normalization at Metabolon, Inc.*

The informatics system consisted of four major components, the Laboratory Information Management System (LIMS), the data extraction and peak-identification software, data processing tools for QC and compound identification, and a collection of information interpretation and visualization tools for use by data analysts. The hardware and software foundations for these informatics components were the LAN backbone, and a database server running Oracle 10.2.0.1 Enterprise Edition.

The purpose of the Metabolon LIMS system was to enable fully auditable laboratory automation through a secure, easy to use, and highly specialized system. The scope of the Metabolon LIMS system encompasses sample accessioning, sample preparation and instrumental analysis and reporting and advanced data analysis. All of the subsequent software systems are grounded in the LIMS data structures. It has been modified to leverage and interface with the in-house information extraction and data visualization systems, as well as third party instrumentation and data analysis software.

Raw data was extracted, peak-identified and QC processed using Metabolon’s hardware and software. These systems are built on a web-service platform utilizing Microsoft’s .NET technologies, which run on high-performance application servers and fiber-channel storage arrays in clusters to provide active failover and load-balancing. Metabolon maintains a library based on authenticated standards that contains the retention time/index (RI), mass to charge ratio (m/z), and chromatographic data (including MS/MS spectral data) on all molecules present in the library. Furthermore, biochemical identifications are based on three criteria: retention index within a narrow RI window of the proposed identification, accurate mass match to the library +/- 10 ppm, and the MS/MS forward and reverse scores between the experimental data and authentic standards. The MS/MS scores are based on a comparison of the ions present in the experimental spectrum to the ions present in the library spectrum. While there may be similarities between these molecules based on one of these factors, the use of all three data points can be utilized to distinguish and differentiate biochemicals. More than 3300 commercially available purified standard compounds have been acquired and registered into LIMS for analysis on all platforms for determination of their analytical characteristics. Additional mass spectral entries have been created for structurally unnamed biochemicals, which have been identified by virtue of their recurrent nature (both chromatographic and mass spectral). These compounds have the potential to be identified by future acquisition of a matching purified standard or by classical structural analysis.

A variety of curation procedures were carried out to ensure that a high quality data set was made available for statistical analysis and data interpretation. The QC and curation processes were designed to ensure accurate and consistent identification of true chemical entities, and to remove those representing system artifacts, mis-assignments, and background noise. Metabolon data analysts use proprietary visualization and interpretation software to confirm the consistency of peak identification among the various samples. Library matches for each compound were checked for each sample and corrected if necessary.

*2.7 Further characterization of historic calculus by GC-MS and UPLC-MS/MS*

Five historic dental calculus samples were pulverization with a pestle and then 15 mg was decalcified with 100 uL of 4% Formic acid at 4˚C for 12 days. Next, 75 uL of 1 M ammonium hydroxide was added, then samples were extracted with 350 uL MeOH + 350 uL Acetonitrile (final 2:2:1 Methanol:Acetonitrile:Water). The extraction solvents differ from samples processed at Metabolon due to independent sample prep optimization; however, no direct comparison has been performed between the different extraction procedures.

For GC-MS analysis, dried extract was derivatized for 90 min with 20 mg/mL methoxyamine hydrochloride in pyridine at 20°C (10 uL) and then with MSTFA for 30 min at 37°C (10 uL). Samples were analyzed by GC-Orbitrap; 1 uL of sample, split 1:10, was injected onto a TraceGOLD TG-5SilMS GC column (cat. no. 26096-1420, Thermo Scientific). Temperature was held at 50°C for 1 min, then ramped to 320°C at rate of 11°C/min, then held at 320°C for 4.40 min. Molecules were analyzed with positive electron-impact (EI)-Orbitrap full scan of 50-650 m/z range.

Raw files were analyzed using Thermo Scientific’s Tracefinder 4.0 deconvolution plugin and unknown screening quantification tool. Deconvolved peaks were searched against NIST 2014 and in-house high-resolution GC libraries; retention index was used to filter search hits. A single quant-ion was used for quantification of deconvolved peaks. Quantified peaks in samples were included if they were at least 10-fold greater than peaks quantified in solvent blanks.

For lipid analysis, dried extract was resuspended in 65:30:5 isopropanol:acetonitrile:water. Lipid LC-MS analysis was performed on a Water’s Acquity UPLC CSH C18 Column (2.1 mm x 100 mm) with a 5 mm VanGuard Pre-Column Mobile coupled to a Q Exactive Focus. Mobile phase A consisted of 70% acetonitrile and 30% water with 10 mM Ammonium acetate and 0.025% acetic acid. Mobile phase B consisted of 90% isopropanol and 10% acetonitrile with 10 mM ammonium acetate and 0.025% acetic acid. Samples were separated using the following 30 min gradient: 2% B for 2 min (0.4 mL/min), increased to 30% B over next 3 min (0.4 mL/min), increased to 50% B over next 1 min (0.4 mL/min), increased to 85% B over next 14 min (0.4 mL/min), increased to 99% B over next 1 min and held at 99% B for 7 min (0.3 mL/min); then returned to 2% to equilibrate for 2 min (0.4 mL/min). Samples were analyzed on the previous gradient using in first positive ion mode and then negative ion mode electrospray ionization (ESI) with full scan MS1 (150-1600 Th) collected 17,000 resolving power (at 400 m/z) for 0-30 min and top-2 data dependent MS2 scans fragmented with stepped normalized collision energy (20-40%).

Raw files were quantified using the Thermo Compound Discoverer^TM^ 2.0 application with peak detection, retention time alignment, and gap filling. Only peaks 10-fold greater than solvent blanks were included in the later analysis. Identification was aided by in-house software and lipid libraries; only compounds with definitive MS/MS evidence were assigned with identification.

UPLC-MS/MS files were further processed through Global Natural Products Social Molecular Networking (GNPS). GNPS settings were kept at the default settings, including MS-clustering (Frank et al. 2011), except for Network options were set to 0.8 Min Pairs Cos and 3 Minimum Matched Fragment Ions, and Library Search options were set to 4 Library Search Min Matched Peaks and 0.8 Score Threshold. The search included all available libraries as of September 14, 2017 (https://gnps.ucsd.edu). Results were filtered for clusters containing one identification hit, clusters with spectra found in 4 of 5 raw files, and clusters containing between 1 and 20 spectra.

**3. References**

Frank, A. M., Monroe, M. E., Shah, A. R., Carver, J. J., Bandeira, N., Moore, R. J., et al. (2011). Spectral archives: extending spectral libraries to analyze both identified and unidentified spectra. *Nature Methods*, *8*(7), 587–591. doi:10.1038/nmeth.1609

Langmead, B., & Salzberg, S. L. (2012). Fast gapped-read alignment with Bowtie 2. *Nature Methods*, *9*(4), 357–359. doi:10.1038/nmeth.1923

Lindgreen, S. (2012). AdapterRemoval: easy cleaning of next-generation sequencing reads. *BMC Research Notes*, *5*(1), 337. doi:10.1186/1756-0500-5-337

Meyer, M., & Kircher, M. (2010). Illumina sequencing library preparation for highly multiplexed target capture and sequencing. *Cold Spring Harbor Protocols*, *2010*(6), pdb.prot5448–pdb.prot5448. doi:10.1101/pdb.prot5448

Ziesemer, K. A., Mann, A. E., Sankaranarayanan, K., Schroeder, H., Ozga, A. T., Brandt, B. W., et al. (2015). Intrinsic challenges in ancient microbiome reconstruction using 16S rRNA gene amplification. *Scientific Reports*, *5*, 16498–19. doi:10.1038/srep16498
